# Investigating the Synergistic Neuroprotective Effects of Plant-Derived Antioxidants and the Psychedelic N,N-Dimethyltryptamine in Alzheimer’s Disease Therapy

**DOI:** 10.3390/cells14120934

**Published:** 2025-06-19

**Authors:** Júlia Jarne-Ferrer, Mercè Pallàs, Christian Griñán-Ferré, Aina Bellver-Sanchis

**Affiliations:** 1 Department of Pharmacology and Therapeutic Chemistry, Institut de Neurociències, Universitat de Barcelona, Avda. Joan XXIII, 27, 08028 Barcelona, Spain; jfjulia97@gmail.com (J.J.-F.); pallas@ub.edu (M.P.); 2 Spanish Biomedical Research Center in Neurodegenerative Diseases (CIBERNED), Instituto de Salud Carlos III, 28029 Madrid, Spain

**Keywords:** Alzheimer’s disease, bioactive compounds, psychedelics, oxidative stress, *Caenorhabditis elegans*

## Abstract

Alzheimer’s disease (AD) is a chronic and complex neurodegenerative disorder characterized by progressive cognitive decline, memory loss, and irreversible impairment of brain functions. The etiology of AD is multifactorial, involving a complex interplay of genetic, environmental, and physiological factors, including the aggregation of amyloid-β (Aβ) and oxidative stress (OS). The role of OS in AD pathogenesis is of particular significance, given that an imbalance between oxidants and antioxidants promotes cellular damage, exacerbates Aβ deposition, and leads to cognitive deterioration. Despite extensive research, current therapeutic strategies have largely failed, likely due to the use of single-target drugs unable to halt the multifactorial progression of the disease. In this study, we investigated the synergistic therapeutic effect of plant-derived bioactive compounds Withanone, Apigenin, Bacoside A, Baicalin, and Thymoquinone in combination with N,N-Dimethyltryptamine (NN-DMT), a psychedelic molecule. We used a transgenic *Caenorhabditis elegans* model to assess the behavioral and molecular outcomes following compound exposure. Motility assays, thioflavin S staining, and survival assays under oxidative stress were employed to evaluate the treatment efficacy. The results of the behavioral and molecular analyses indicated that the combination therapy exhibited a higher efficacy than the monotherapies, leading to a significant reduction in age-related motility defects in the AD model. Furthermore, the combination treatment substantially reduced Aβ plaque burden, enhanced survival following OS insult, and demonstrated a synergistic effect in mitigating AD-related hallmarks. Taken together, these findings support the potential of combining NN-DMT with specific bioactive compounds as a promising multi-target therapeutic approach for AD.

## 1. Introduction

Alzheimer’s disease (AD) is the most prevalent cause of dementia [1], with major impacts among the elderly, age being an important risk factor. AD is a chronic, progressive neurodegenerative disorder characterized by memory loss, cognitive decline, and behavioral changes [2]. The etiology of AD appears to be complex and multifactorial [3]. While it is primarily characterized by the abnormal accumulation and deposition of β-amyloid (Aβ) peptides into extracellular plaques [4,5] and the hyperphosphorylation of tau [4,6], other factors, including neuronal loss [7,8], neuroinflammation [9,10], and oxidative stress (OS) [11,12], also play a crucial role in the pathogenesis. Of interest, it has been demonstrated a direct link between AD pathology and OS in in vitro and in vivo studies [13,14,15,16]. OS results from an imbalance between reactive oxygen species (ROS) production and the ability to neutralize them through antioxidant defenses, leading to cumulative cellular damage [17]. The overproduction of ROS can damage the central nervous system (CNS) by oxygen modification of macromolecules such as lipids, proteins, and nucleic acids [18]. This process is further exacerbated by the age-related decline in antioxidant enzyme activity, which disrupts synaptic activity, ultimately contributing to cognitive dysfunction [18,19,20]. Additionally, evidence indicates that the accumulation of Aβ exacerbates mitochondrial dysfunction and ROS production, creating a vicious cycle that amplifies neuronal damage and cognitive decline [21].

Due to the multifaceted nature of the disease, the absence of a single-target therapy capable of halting or reversing its progression has emerged as a significant challenge [22,23,24]. Given this challenge, a promising avenue for more effective treatment strategies involves the combination of therapies that address multiple pathogenic pathways, including OS and Aβ aggregation, among others [22]. Thus, employing this approach can potentially enhance disease management and improve cognitive outcomes. Increasing attention has been given to non-pharmacological interventions as potential strategies to prevent AD or slow its progression. In particular, nutraceuticals have been widely recognized in the literature for their potential to mitigate the risk and progression of various neurodegenerative diseases, including AD [25].

Natural bioactive compounds derived from plants have shown great promise as therapeutic candidates due to their antioxidant, anti-inflammatory, and neuroprotective properties. However, there is a lack of evidence to support the efficacy of these substances in humans. Examples of this include Withanone (*Withania somnifera*) [26,27,28,29,30,31], which promotes proteostasis and neuronal differentiation, and Apigenin (found in parsley, chamomile, and celery) [32,33,34,35,36], which has demonstrated anti-inflammatory and antioxidant activity via modulation of the NRF2 pathway. Moreover, evidence has demonstrated that Bacoside A (*Bacopa monnieri)* [37,38,39] enhances synaptic plasticity and mitigates Aβ-induced toxicity, while Baicalin (*Scutellaria baicalensis*) [40,41,42] exhibits a reduction in oxidative damage and apoptosis. Furthermore, Thymoquinone (*Nigella sativa*) [43,44,45] has been shown to exhibit mitochondrial protective properties and anti-inflammatory action. Although clinical trials have been conducted to study their effects [46,47,48,49,50,51,52,53] on neurodegenerative disorders, further research is necessary to fully understand their potential impact on these conditions. Furthermore, it is important to note that most clinical studies have evaluated cognitive function in healthy subjects, which may introduce a potential bias when applying these findings to patients with neurodegenerative diseases.

In addition to these bioactive compounds, emerging evidence suggests that psychedelic substances may also hold potential as therapeutic agents for neurodegenerative diseases, although their use is still in the early stages. One example is N,N-Dimethyltryptamine (NN-DMT), a natural hallucinogenic alkaloid richly existing in Ayahuasca, whose clinical application is complicated due to regulatory and safety concerns [54]. NN-DMT has a very high affinity for 5HT1A receptors [55] and is gaining attention for its unique neuroplastic and immune-modulatory properties, which could contribute to neurodegeneration and resilience [56,57]. However, given that NN-DMT primarily exerts its effects via serotonergic receptors, it remains unclear whether its chronic use could induce desensitization or receptor downregulation, potentially limiting long-term efficacy. Moreover, no studies have observed direct effects of NN-DMT on OS, a critical factor in AD pathology.

The combination of active ingredients, such as bioactive compounds and NN-DMT, has the potential to produce additive or synergistic effects, thereby enhancing therapeutic outcomes with minimal side effects [58,59]. However, studies investigating the synergistic effects of these compounds for AD treatment remain scarce. Therefore, this study aims to evaluate the combined therapeutic potential of bioactive compounds and NN-DMT on key pathological features of AD, particularly Aβ deposition and OS. To this end, we employed a transgenic *C. elegans* model of AD and utilized behavioral and molecular analyses to assess the effects of these compounds, both individually and in combination. The *C. elegans* is a genetically tractable and rapid screening platform for identifying compounds that modulate amyloid-related toxicity. While it does not fully recapitulate mammalian neuronal architecture or cognitive decline, it reliably models key biochemical and cellular hallmarks of AD, making it a valuable initial screening tool. [60]. This study’s findings underscore the potential of combination therapies in addressing the multifactorial nature of AD, thereby laying the groundwork for novel strategies to combat this debilitating disease.

## 2. Methods

### 2.1. Compounds

Stock concentrations of natural extracts: Whitanone (TM-TN5259, CAS#27570-38-3, Targetmol, Boston, MA, USA), Bacoside A (PHL89576, CAS#11028-00-5, Merck, Rahway, NJ, USA), Thimoquinone (T0795, CAS#490-91-5, TCI, Tokyo, Japan), Apigenin (1102S, CAS#520-36-5, Extrasynthese, Genay, France), Baicalin (1280S, CAS#21967-41-9, Extrasynthese), and the NN-DMT (SML0791, CAS#61-50-7, Sigma-Aldrich, Sant Louis, MO, USA) were prepared in 100% dimethyl sulfoxide (DMSO). Then, stock solutions were diluted in MiliQ ddH_2_O, obtaining the tested dilutions (1, 5, 10, 25, and 50 µM) at a maximum concentration of DMSO 1%, and stored at −20 °C.

### 2.2. C. elegans Maintenance and Treatment

The wild-type *Caenorhabditis elegans* (*C. elegans*) strain N2 and the AD transgenic strain CL2006 were used. Standard methods were used for culturing and observing *C. elegans* [61]. N2 was maintained at 20 °C, while CL2006 was propagated at 16 °C in a temperature-controlled incubator on a solid nematode growth medium (NGM) seeded with *Escherichia coli* (*E. coli*) OP50 strain as a food source. To obtain the age-synchronized population of eggs, gravid adults were treated with alkaline hypochlorite solution (0.5 M NaOH, ∼2.6% NaCl) for 5–7 min. Eggs were suspended in S-medium, and L1 larvae were allowed to hatch overnight without food.

**Figure 1:**
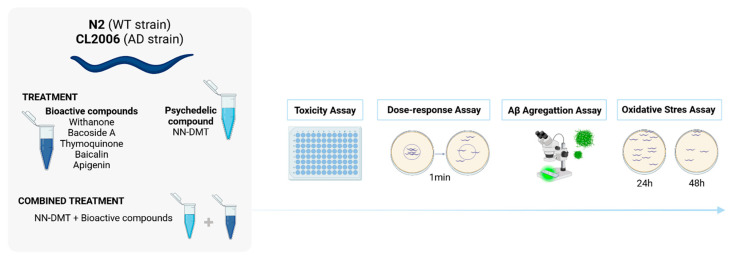
Experimental design used for this study. After synchronized hatching, L1 larvae of the CL2006 strain were treated with individual compounds or combinations in 96-well plates for four days. Assays performed included food clearance to assess general toxicity and developmental viability, motility assay to monitor age-related paralysis, Thioflavin-S staining to quantify Aβ aggregation, and oxidative stress survival assay following tert-butyl hydroperoxide exposure. All analyses were performed using standardized protocols.

Drug assays were performed in a 96-well plate format in liquid culture to quantify food clearance, OS, and amyloid aggregation, and treated for 4 days at 20 °C. Each well contained a final volume of 60 µL, including approximately 25–30 L1 stage worms, the tested compound(s) at the appropriate concentration, and heat-inactivated *E. coli* (OD595 = 0.8–0.9), which served as a food source. A schematic diagram of the experimental design is shown in Figure 1.

### 2.3. Food Clearance Assay

N2 (WT) worms were subjected to a drug assay as described above. However, for this assay, the OD_595_ of the inactivated OP50 bacteria was adjusted to 0.7 A.U.). Worms were grown with continuous shaking at 180 rpm at 20 °C for 7 days. Tested concentrations ranged between 1 and 50 µM. For control wells, 1% DMSO (non-toxic concentration) and 5% DMSO (toxic concentration) were used; blank wells were used with S-medium and S-medium complete only, without eggs or inactivated OP50, respectively. The OD595 was measured daily, which is an indicator of the effect of compounds on *C. elegans.* It was monitored by the rate at which the OP50 suspension was consumed as a readout for *C. elegans* growth, survival, or fertility.

### 2.4. Thioflavin-S Staining Aß Aggregation

After treatment, adult CL2006 worms were in 4% paraformaldehyde/phosphate-buffered saline (PBS), pH 7.5, for 24 h at 4 °C and permeabilized in 5% fresh β-mercaptoethanol, 1% Triton X-100, and 125 mm Tris, pH 7.5, at 37 °C for another 24 h. All reagents were freshly prepared. CL2006, treated or untreated, was stained with 0.125% thioflavin S (Sigma, Sant Louis, MO, USA) in 50% ethanol for 2 min, destained in 50% EtOH for 2 min, washed 3 times with PBS, and transferred in approximately 10 µL volume on a droplet of Fluoromount G on a glass slide for microscopy. Fluorescence images were acquired using a 20 Å objective of a fluorescence microscope. Amyloid deposits in the head region of worms were quantified by counting the number of Thioflavin S (ThS)-positive spots using ImageJ2 2.16.0 and were expressed as amyloid-β (Aβ) deposits/anterior area. Dye-only controls were used to exclude nonspecific staining or autofluorescence.

### 2.5. Oxidative Tolerance Assay

To investigate sensitivity to oxidative stress after the different treatments, N2 (WT) worms were transferred onto plates that included 6.2 mM tert-butyl hydroperoxide (ThermoFisher, Kander, Germany) in NGM agar. Worms were incubated on these plates at 20 °C for 2 h. Then, worms were transferred to new NGM plates seeded with OP50 and without tert-butyl hydroperoxide. Worms were observed at 48 h post-intervention and were designated as dead when they failed to respond to repeated prodding with a pick.

### 2.6. Statistics

Data analysis was conducted using GraphPad Prism ver. 10.4.1 statistical software. Data are expressed as the mean ± standard error of the mean (SEM) of at least 3 experiments. Means were compared with one-way analysis of variance (ANOVA), followed by the Tukey post hoc test. Comparison between groups was also performed by two-tailed Student’s *t*-test for independent replicates when it was necessary. Statistical significance was considered when *p*-values were <0.05 (* *p* < 0.05; ** *p* < 0.01; *** *p* < 0.001; **** *p* < 0.0001). The statistical outliers were determined with Grubbs’ test and removed from the analysis.

## 3. Results

### 3.1. Natural Bioactive Compounds and NN-DMT Have a Non-Toxic Effect on C. elegans

**Figure 2:**
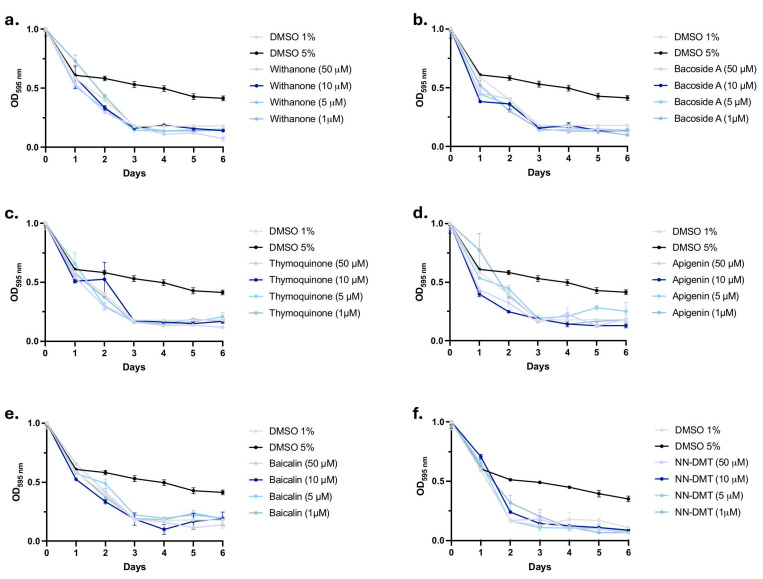
Non-toxic effect of bioactive compounds and NN-DMT in the N2 strain. Food clearance assay assessing the toxicity of the bioactive compounds (**a**) Withanone, (**b**) Bacoside A, (**c**) Thymoquinone, (**d**) Baicalin, and (**e**) Apigenin and of the psychedelic substance (**f**) NN-DMT at 1, 5, 10, and 50 µM concentrations. Results are expressed as the mean ± standard error of the mean (SEM) (*n* = 3). Groups were compared by a non-linear regression model for sigmoidal curves against positive control.

In its food clearance experiment, natural bioactive compounds and NN-DMT potential are evaluated using *C. elegans*, a model organism with a short lifespan that can thrive in liquid cultures of *E. coli* [62]. Withanone, Bacoside A, Thymoquinone, Apigenin, Baicalin, and NN-DMT were administered to *C. elegans* N2 (WT) at 1, 5, 10, and 50 µM concentrations. The goal was to determine if some of these concentrations were toxic to safeguard the *C. elegans* physiology and ability to hatch for further experiments. Figure 2 shows a plot of the OD of the *E. coli* OP50 suspension over the 6 days of the food clearance assay. DMSO 1% corresponded to drug vehicles and was used as a negative (safe) control, whereas DMSO 5% was used as a positive (toxic) control. The four different concentrations of each bioactive compound tested, as well as the NN-DMT (1, 5, 10, and 50 µM), were classified as safe, as the OD decrease paralleled the plot of vehicle control samples, and visual inspection confirmed normal growth of the animals. Then, we used those concentrations to perform the functional experiments (Figure 2).

### 3.2. Impact of Natural Bioactive Compounds and NN-DMT on Locomotor Defects and Aβ Levels Exhibited by an AD Transgenic C. elegans Strain

**Figure 3:**
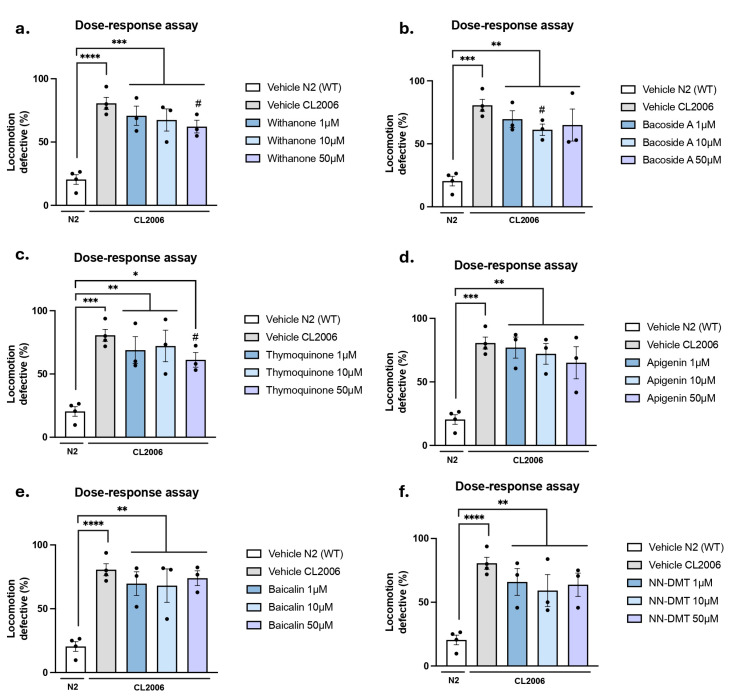
Locomotion defect after bioactive compounds and NN-DMT treatment in the CL2006 strain. Effects of the natural bioactive compounds (**a**) Withanone, (**b**) Bacoside A, (**c**) Thymoquinone, (**d**) Baicalin, and (**e**) Apigenin and the psychedelic substance (**f**) NN-DMT at 1, 10, and 50 µM on the age-dependent paralysis of CL2006 worms. Results are expressed as a mean ± SEM (*n* = 3, 30 worms in each group/replicate). Groups were compared by a one-way ANOVA test, followed by a Tukey post hoc test. For Vehicle CL2006 vs. Whitanone 50 µM, Thymoquinone 50 µM, and Bacoside A 10 µM, data were analyzed using a two-tailed Student’s *t*-test. *# p* < 0.05; * p < 0.05; ** p < 0.01; *** p < 0.001; **** p < 0.0001.

The AD transgenic *C. elegans* strain known as CL2006, which expresses human Aβ₁₋₄₂ in body wall muscle cells under the control of the unc-54 promoter, leading to progressive age-dependent paralysis that begins during early adulthood and worsens with age due to Aβ aggregation and associated cellular toxicity [63]. Due to these characteristics, this model reliably mimics the key pathological features of AD, including impaired motor function and β-amyloid accumulation. Therefore, a motility assay was conducted to evaluate motor function, in which worms were placed at the center of a plate with food and allowed to explore freely for one minute. The N2 (WT) and CL2006 strains were treated with 1% DMSO as a drug vehicle. As expected, the locomotion defect of the percentage of WT worms was lower than CL2006 due to age-dependent paralysis. In general, none of the treatments with the natural bioactive compounds and NN-DMT could impact the locomotion exhibited by the CL2006 compared to the control group (Figure 3). However, in the case of the Withanone and the Thymoquinone (Figure 3a,c) at the highest dose of 50 µM and the Bacoside A (Figure 3b) at the 10 µM dose, a slightly significant reduction in the percentage of locomotion defects was observed.

**Figure 4:**
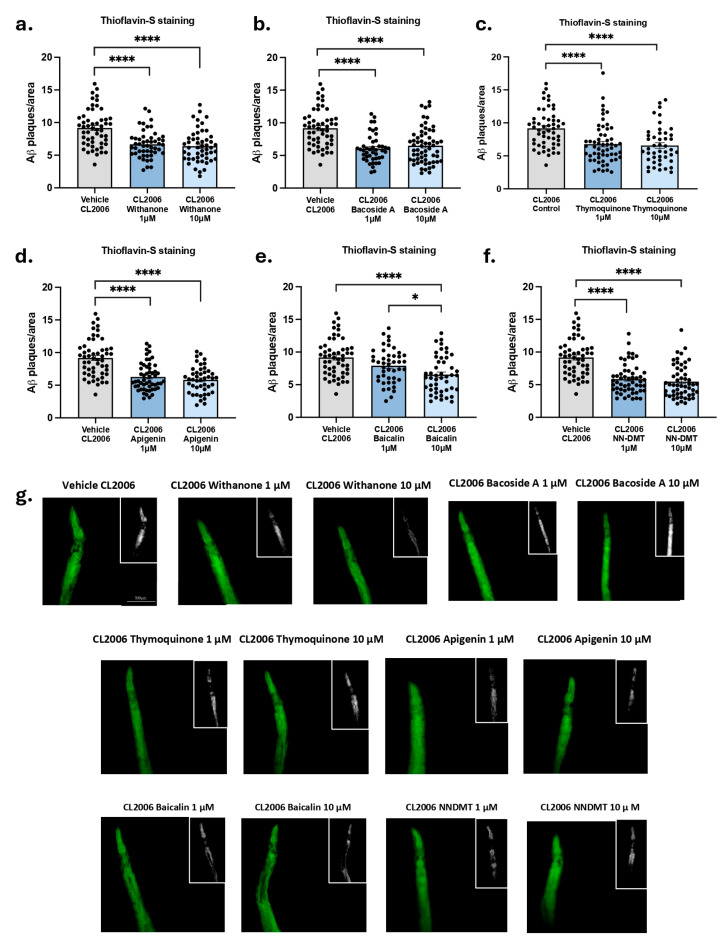
Aβ plaques after bioactive compounds and NN-DMT treatment in the CL2006 strain. Quantification of Thioflavin S-positive particles in the head region of the CL2006 strain after (**a**) Withanone, (**b**) Bacoside A, (**c**) Thymoquinone, (**d**) Baicalin, (**e**) Apigenin, and (**f**) NN-DMT treatment at 1 µM and 10 µM concentrations. (**g**) Representative images from each group tested. Results are expressed as a mean ± SEM (*n* = 3, 10 worms in each group/replicate). Groups were compared by a one-way ANOVA test, followed by Tukey post hoc analysis. White boxed insets show magnified views of Aβ-positive puncta used for quantification. * p < 0.05; **** p < 0.0001.

Next, we scored the number of Aβ deposit levels in the head of the CL2006 to evaluate the effect of each individual compound on the reduction in these levels. Contrary to the observations made in the locomotion defective experiment, Figure 4 demonstrates that all compounds exhibited a substantial impact on Aβ_1-42_ peptide deposits at both lower concentrations (1 µM and 10 µM), except the Baicalin treatment (Figure 4e,g), which exhibited a significant reduction in Aβ levels only at the 10 µM concentration.

### 3.3. Oxidative Stress Tolerance and Lifespan Modulation in C. elegans with Natural Bioactive Compounds While NN-DMT Lacks Antioxidant Effects

**Figure 5:**
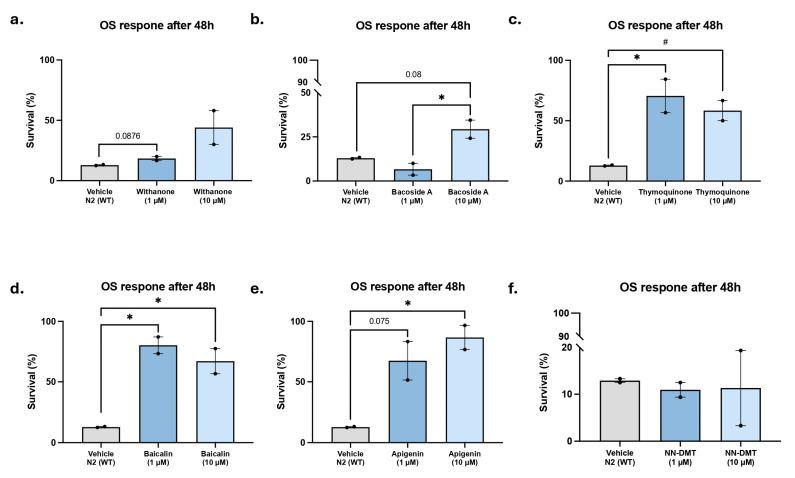
Survival percentage after tert-butyl exposure in the N2 strain. Survival percentage of N2 worms treated with (**a**) Withanone, (**b**) Bacoside A, (**c**) Thymoquinone, (**d**) Baicalin, (**e**) Apigenin, and (**f**) NN-DMT at 1 µM and 10 µM concentration after 48 h of tert-butyl exposure. Results are expressed as a mean ± SEM (*n* = 2, 30 worms in each group/replicate). Groups were compared by a one-way ANOVA test, followed by Tukey post hoc analysis. *# p* < 0.05; * p < 0.05.

OS has been demonstrated to be associated with the etiology of age-related cognitive decline and AD [64], promoting senescence [65,66]. Accordingly, it has been demonstrated that OS insults impact the lifespan of *C. elegans*, reducing it [67,68]. In light of these findings, an oxidative tolerance assay was conducted on the N2 (WT) *C. elegans* strain, and the treatment effect on lifespan was observed up to 24 and 48 h after exposure to tert-butyl (6.2 mM), a chemical oxidant. Exposure to 6.2 mM tert-butyl hydroperoxide reduced survival by approximately 50% after 48 h in untreated N2 worms, confirming the sensitivity of the assay to oxidative stress. The natural bioactive compounds generally exhibited an antioxidant effect at a concentration of 10 µM (Figure 5c–e). Furthermore, a significant increase in survival was observed after tert-butyl exposure at a concentration of 1 µM, following treatment with Thymoquinone and Baicalin (Figure 5b,c) from 48.3% (control) to 68.4% survival and to 70.1%, respectively. Similarly, a favorable trend towards enhanced worm survival was observed following Bacoside A treatment at 10 µM and Whitanone and Apigenin treatment at 1 µM (Figure 5a,e). Lastly, the NN-DMT does not demonstrate any antioxidant properties at both tested concentrations (Figure 5f).

### 3.4. Synergistic and Additive Effects of Natural Bioactive Compounds and NN-DMT on Locomotion, Aβ Aggregation, and OS Responses in C. elegans

**Figure 6:**
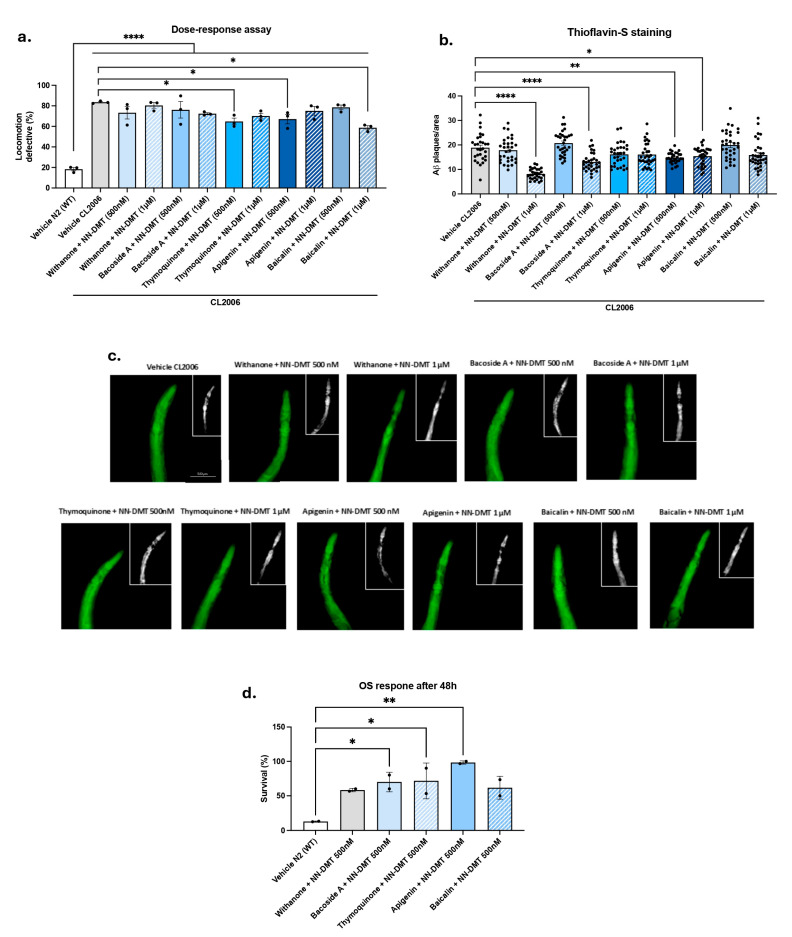
Synergistic and additive effects after the combined treatments. Motility effect of NN-DMT treatment in combination with (**a**) Withanone, Bacoside A, Thymoquinone, Baicalin, and Apigenin on CL2006 worms’ age-dependent paralysis at 1 µM and 500 nM concentrations. (**b**) Quantification of Thioflavin S-positive particles in the head region of the CL2006 strain after combining NN-DMT with the different bioactive compounds at 1 µM and 500 nM. (**c**) Representative images of Aβ plaques stained with Thioflavin-S from each group tested. White boxed insets show magnified views of Aβ-positive puncta used for quantification. (**d**) Survival percentage of N2 worms treated with the specific combinations at 500 nM concentration after 48 h of tert-butyl exposure. Results are expressed as a mean ± SEM (*n* = 3, 30 worms in each group/replicate for motility assay; *n* = 3, 10 worms in each group/replicate for Thioflavin-S assay; and *n* = 2, 30 worms in each group/replicate for tert-butyl assay). Groups were compared by a one-way ANOVA test, followed by Tukey post hoc analysis. * p < 0.05; ** p < 0.01; **** p < 0.0001.

As previously mentioned, combinations of different active ingredients in extracts may result in additive or synergistic effects, thereby enhancing disease-modifying activity [59]. Given that each compound appears to have beneficial effects in the AD strain *C. elegans* at high concentrations, we investigated whether combining different natural bioactive compounds and NN-DMT would produce synergistic or additive effects. To this end, the impacts of combinations of Withanone, Bacoside A, Thymoquinone, Apigenin, and Baicalin with NN-DMT on locomotion defects and Aβ levels were examined at two low concentrations, i.e., 1 µM and 500 nM. The Thymoquinone + NN-DMT 500 nM combination exhibited an additive effect on locomotion defects. Furthermore, the Apigenin + NN-DMT 500 nM combination demonstrated a synergistic effect in reducing locomotive defects. In the case of the combination Baicalin + NN-DMT 500 nM, the amelioration of the locomotion defect was observed at the highest concentration (1 µM) due to its synergistic effect (GraphPad Prism ver. 10.4.1). Regarding the Thioflavin-S (ThS) staining, additive effects reducing Aβ aggregation levels were obtained after Withanone + NN-DMT 1 µM and Bacoside A + NN-DMT 1 µM. A notable observation was the occurrence of an additive effect in the treatment combining Apigenin and NN-DMT, as evidenced by a significant reduction in Aβ aggregation levels at both concentrations (1 µM and 500 nM) (Figure 6b,c).

It is noteworthy that, despite the extant literature’s characterization of NN-DMT as a potential treatment for AD [55,57,69], there is a lack of evidence regarding its impact on OS responses, an important contributing factor of the etiopathogenesis of AD. Consequently, the rationale for the dual approach proposed in this study, which involves the combination of natural bioactive compounds and NN-DMT, is to take advantage of the established antioxidant effect of the natural bioactive compounds. A positive synergistic effect was observed after treatment with Bacoside A + NN-DMT, Thymoquinone + NN-DMT, and Apigenin + NN-DMT at 500 nM, which increased the survival percentage after tert-butyl exposure in comparison with the control group (Figure 6d).

## 4. Discussion

AD is a complex neurodegenerative disorder characterized by progressive cognitive decline and remains without a cure. Given their multifactorial nature, therapeutic strategies targeting multiple pathological mechanisms have gained increasing interest. A growing body of evidence suggests that both natural bioactive compounds [70] and psychedelic compounds [69,71,72] may mitigate AD-related pathology through antioxidant, anti-inflammatory, and anti-aggregative mechanisms. In this context, we observed that Thymoquinone, Withanone, and Bacoside A positively affected Aβ aggregation and OS tolerance. However, a significant challenge is their limited bioavailability. For instance, Thymoquinone exhibits poor solubility and is rapidly metabolized, hindering its ability to reach therapeutic levels in the brain [73]. A similar challenge is faced by Withanone and Bacoside A, which exhibit poor absorption and, consequently, limited therapeutic efficacy in humans [74,75]. Consequently, while these compounds have exhibited positive effects in animal models, the clinical evidence remains inconsistent. For instance, large-scale trials for Bacoside A have not consistently replicated cognitive improvements [75], and Withanone and Thymoquinone have yet to meet the required clinical endpoints. To overcome bioavailability limitations, emerging strategies include nanoformulation, liposomal encapsulation, and conjugation to brain-targeting peptides. For example, Apigenin-loaded nanoparticles have shown a reduction in inflammation in murine models, and lipid-based carriers enhance and Thymoquinone-loaded chitosan nanoparticles enhance stability and absorption [76,77].

On the other hand, the use of psychedelics such as NN-DMT in neurodegenerative diseases like AD also presents significant challenges. One major concern is the potential for serotonergic receptor desensitization, which could lead to diminished efficacy over prolonged use. Chronic activation of 5-HT2A receptors, the primary target of NN-DMT, may result in receptor downregulation, potentially attenuating its long-term neuroprotective effects [54]. Additionally, regulatory and safety concerns pose substantial barriers, as the psychoactive nature of NN-DMT necessitates stringent monitoring and careful patient selection. Its long-term effects on brain function, particularly in elderly populations with neurodegenerative disorders, remain poorly understood and require further investigation through rigorous clinical trials.

Monotherapy approaches have historically been the mainstay of AD treatment, but their efficacy is often limited by factors such as patient response variability, drug resistance, and potential toxicity [24]. In contrast, combination therapies, which involve the simultaneous use of multiple agents targeting different pathological pathways, have emerged as a promising alternative. These strategies have the potential to enhance treatment efficacy, minimize side effects, and reduce the likelihood of resistance, thereby offering a more comprehensive approach to managing AD’s complexity [78]. While previous studies have investigated the individual effects of NN-DMT on neuroplasticity, little research has explored its potential synergistic interactions with natural antioxidants. Addressing this gap, our study investigated the combination of natural bioactive compounds with NN-DMT to assess their effects on key AD features, such as Aβ aggregation and oxidative stress, using a transgenic *C. elegans* model.

The findings of this study demonstrated that the combination of Apigenin and NN-DMT produced a synergistic reduction in locomotor defects, while Thymoquinone and NN-DMT exhibited additive effects. This finding aligns with previous research suggesting that Apigenin exerts neuroprotective effects by modulating oxidative stress and neuroinflammation via the Nrf2 pathway [79]. Furthermore, NN-DMT has been demonstrated to stimulate neuroplasticity by activating 5-HT2A receptors [80], which could enhance the efficacy of bioactive compounds by promoting neuronal resilience. Thioflavin S (ThS) staining revealed that bioactive compounds significantly reduced Aβ aggregation, with Withanone and Apigenin showing the most robust effects. Previous research has demonstrated that Withanone reduces amyloidogenic processes by stabilizing cellular proteostasis and activating chaperone-mediated autophagy [81,82]. Notably, NN-DMT alone did not exhibit antioxidant effects, which is consistent with studies indicating that NN-DMT primarily acts through serotonergic pathways with limited direct influence on redox homeostasis [55]. However, combining NN-DMT with bioactive compounds resulted in enhanced survival in oxidative stress assays, suggesting an indirect role in modulating cellular resilience. The collective findings of these studies suggest that NN-DMT may enhance the bioactivity of natural compounds through complementary mechanisms, possibly involving the upregulation of brain-derived neurotrophic factor (BDNF) and the modulation of inflammatory pathways [55]. Given the established role of neuroinflammation and OS in the pathophysiology of AD, the observed synergy between NN-DMT and bioactive compounds may present a novel therapeutic approach to address this complex condition.

While the findings of this study provide promising insights into the potential therapeutic synergy between natural bioactive compounds and NN-DMT in an AD model, some limitations must be acknowledged. First, bioavailability and pharmacokinetics of the tested compounds in humans may differ significantly, limiting direct translational potential. Second, the precise mechanisms underlying the observed synergistic effects remain to be elucidated, particularly at the receptor and signaling pathway levels. Third, while statistical significance was achieved for several treatments, some variability was observed, particularly in oxidative stress assays, as reflected by large SEMs. This can be attributed to inter-assay variation and natural variability in worm responses. Nevertheless, consistent trends across independent replicates confirm the reliability of the findings. These limitations highlight the need for future studies using mammalian models and clinical investigations.

In summary, the present study supports the potential therapeutic benefits of combining NN-DMT with natural bioactive compounds in targeting multiple AD pathophysiological mechanisms. The observed synergistic effects on locomotion, Aβ aggregation, and OS suggest that combination therapies could provide a more effective treatment strategy for AD compared to monotherapies. Future research should focus on elucidating the precise molecular pathway between NN-DMT and these natural bioactive compounds that mediates these synergistic effects, such as BDNF signaling and mitochondrial regulation. Likewise, test these combinations in mammalian models to assess pharmacokinetics, safety, and cognitive outcomes.

## Data Availability

The datasets used, generated, and/or analyzed during the current study are available from the corresponding author upon reasonable request.
